# A case report of usual presentation of unusual bladder mass-polypoid cystitis in a young Indian female

**DOI:** 10.1016/j.ijscr.2024.110640

**Published:** 2024-11-22

**Authors:** Sajal Gupta, Prashanth Adiga K., Kishan Raj K., Nandakishore Bhat

**Affiliations:** Department of Urology & Renal Transplantation, Father Muller Medical College, Mangalore, Karnataka 575002, India

**Keywords:** Lower urinary tract symptoms (LUTS), Polypoid cystitis, Trans urethral resection of bladder tumor (TURBT), von Brunn nests

## Abstract

**Introduction and importance:**

Polypoid cystitis is a rare benign exophytic lesion affecting the bladder mucosa that clinically and radiologically resembles urothelial carcinoma. An adequate diagnosis of this pathology requires histological evaluation. Owing to the rare occurrence of this benign urinary bladder lesion, its prevalence is under-reported, with very few cases reported.

**Case presentation:**

Here, we report a case of polypoid cystitis in a young Indian female, who presented to the urology department with obstructive LUTS and a history of catheterization for urinary retention. The patient was evaluated and found to have a usual bladder mass that was clinically and radiologically suspected to be a carcinoma bladder, so TURBT of the mass was performed. The mass was completely resected and sent for HPE, which revealed features of polypoid cystitis.

**Clinical discussion:**

Polypoid cystitis should be considered in the differential diagnosis of urinary bladder masses presenting at a younger age with a history of dysuria, LUTS, multiple catheterizations for retention of urine along with no history of hematuria and negative familial history for carcinoma. One of the common factors in all cases of polypoid cystitis is a history of long-standing catheterization, which might predispose patients to such a benign lesion or vice versa. Adequate histopathological evaluation of these benign lesions is crucial for accurate diagnosis.

**Conclusion:**

Therefore, in young female/males without a familial history of bladder carcinoma or environmental carcinogen exposure, benign bladder lesions should be suspected first rather than malignant lesions, although malignant lesions of the bladder are far more common than benign lesions.

## Highlights

• This is an usual presentation of benign bladder lesion which is itself unusual.
• Clinical and radiological presentation of the case suggests of malignant lesion but turns out to be benign lesion.
• Polypoidal cystitis can become aggressive and obstructive.
• Its presentation in the young age group, females and with urethral involvement is uncommon.
• Though benign but may require aggressive resection and treatment.

## 1 Introduction

Masses within the urinary bladder are common, and the majority of them are diagnosed as malignancy [1]. Bladder cancer is the seventh most common malignancy worldwide and is associated with remarkable mortality and morbidity [2,3]. Among malignant tumors, the most frequently encountered histological subtype is urothelial carcinoma, which accounts for 90 % of all diagnosed bladder cancers [4]. Other types of benign masses arising inside the urinary bladder account for only 1 %–5 % of all masses. These benign lesions are often misdiagnosed as bladder cancer, owing to their similar clinical and radiological characteristics [5,6]. Histopathological evaluation plays a key role in the diagnosis of these lesions. The incidence of benign bladder lesions is underreported owing to their rarity, with very few cases reported in the Indian population. Here, we report a case of polypoid cystitis, a rare benign lesion of the urinary bladder, in a 21-year young Indian female. The motive in reporting this case is to widen the horizon so to keep benign lesions of the bladder in the differential diagnosis.

## 2 Case presentation

**Fig. 1:**
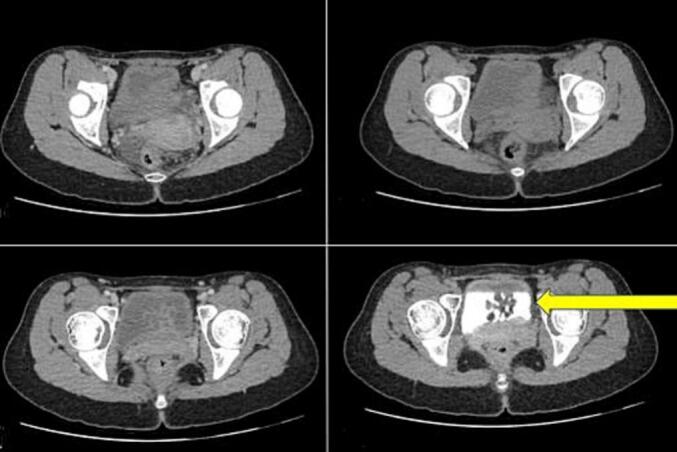
Sequential CT scan images polypoidal lesions arising from the base of urinary bladder and projecting into the lumen.

A 21-year young female patient reported to the Department of Urology with chief complaints of lower urinary tract symptoms(LUTS) for 5 months(mainly dysuria, frequency and urgency), without the history of fever, hematuria, or flank pain. The patient had a history of cystoscopy and transurethral removal of bladder tumor (TURBT) five years back, and a history of catheterization six months back for urinary retention. No abnormalities were detected on general physical examination, per abdominal and external genitalia examination including meatus. CT scan of the abdominal and pelvic region showed a distended bladder and diffuse wall thickness with a maximum thickness of 6.5 mm. Peripherally enhancing broad based lobulated polypoid lesion, measuring (CC × AP × TR) 2.8 × 2.8 × 3.2 cm arising from the base of urinary bladder and projecting into the lumen was seen (Fig. 1).

**Fig. 2:**
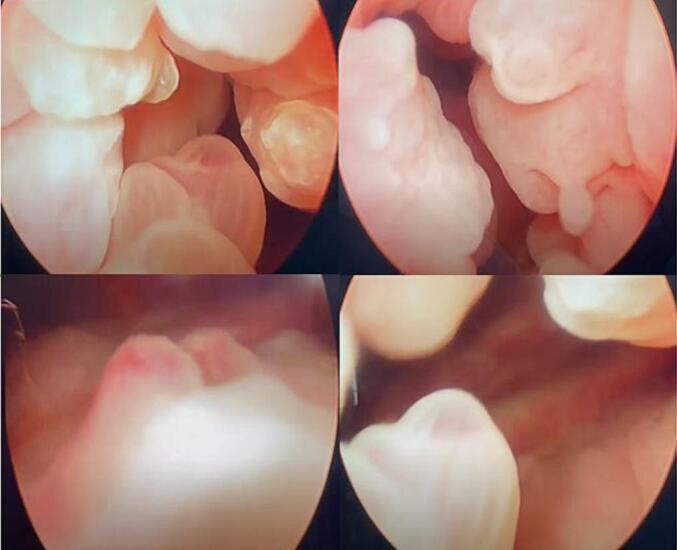
Cystoscopy showing multiple bladder growths arising from bladder neck and urethra at 1, 7, 11 o'clock position along with lobulated lesion at the trigone.

**Fig. 3:**
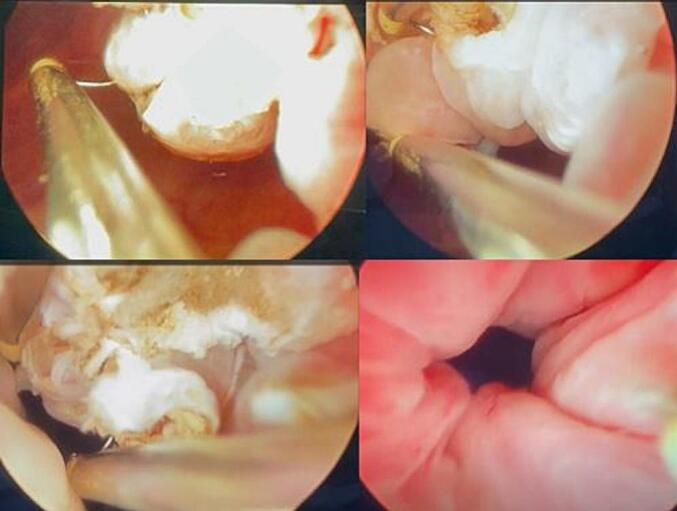
Intraoperative images showing resection of the lesion with post resection completely opened urethra and the bladder neck.

This lesion was present in close proximity to the left vesicoureteric junction, but there was no evidence of upstream dilatation. All routine blood tests, including renal function tests, were within the normal range. No bacterial growth was observed in the urine culture. Urine microscopy with cytology showed urothelial cells in the background of inflammatory infiltrate, bacterial colonies & RBC. There was no evidence of atypical or malignant cells. The patient was planned for cystoscopy, which revealed multiple growths arising from the bladder neck and urethra being proximal to the external sphincter, first measuring 3x4cm, arising from proximal urethra protruding into the bladder at 1′0 clock position, second measuring 2x2cm protruding from proximal urethra into bladder at 11′0 clock, and third 1 × 0.5 cm growth arising from external meatus at 7′0 clock. The rest of the bladder walls, along with the bilateral ureteric orifice, were normal, but the trigone was edematous with multiple lobulated lesions (Fig. 2). A passive bipolar resectoscope was inserted, piecemeal resection of the lesion was performed, and chips were removed using an Ellick evacuator (Fig. 3). Meatoplasty was done for perimeatal lesions.

After complete removal of the external growth, haemostasis was achieved. 18Fr 3 way per urethral catheter (PUC) was placed, and irrigation was started. The excised masses were sent for histopathological evaluation, which showed polypoid fragments of bladder mucosa covered predominantly by stratified squamous epithelium and focally by the urothelium. Hyperplasia of the epithelium was observed with normal maturation. The sub-epithelium showed congested blood vessels and myxoid stroma with a lymphocyte-predominant mixed inflammatory infiltrate. Focal von Brunn nests were also observed. There was no evidence of malignancy. Therefore, the final diagnosis of polypoid cystitis was made. The patient is under regular follow-up without any evidence of recurrence for one year.

The work has been reported in line with the SCARE criteria [19].

## 3 Discussion

Polypoid cystitis, originally described by Mostofi, is a rare, benign lesion of the urinary bladder. It presents as an exophytic polypoid lesion with chronic inflammation and prominent stromal edema [7]. Diagnosis of polypoid cystitis was first made by Friedman and Ash in 1959 [8]. It has been postulated to occur as a result of irritation of the bladder mucosa, particularly in the presence of a urethral catheter [7,9,10]. In our case, there was a history of catheterization six months back. It can also occur in patients with benign prostatic hyperplasia, urethral stones, and bladder stones. Other factors, such as a history of treatment for bladder cancer, ureter carcinoma, or radiation therapy for prostate cancer, may result in polypoid cystitis [8,11]. Characteristic histological features of this pathology were originally described by Ekelund et al. [12] in 40 of 51 patients who were treated with urethral catheterization. The frequency of polypoid cystitis increases with time, with most of the patients presenting with acute retention of urine and catheterization for the same [12]. In our case, the patient had a history of retention and catheterization 6 months back and LUTS with dysuria. Polypoid cystitis can occur over a wide age range from 20 months to 93 years [13,14]. The Mean age of occurrence was 63 years [11]. Based on previously reported cases, males are more commonly affected than females, with a male-to-female ratio of 3:1 [11,13]. However, in the present case, the patient was a 21-year female. The clinical presentations of polypoid cystitis may range from being asymptomatic to bladder obstruction, voiding dysfunction, colovesical fistula, and gross haematuria [11]. Our patient had lower urinary tract symptoms for 5 months. The radiographic findings of polypoid cystitis are not specific and cannot distinguish it from urothelial carcinoma [15]. This condition can be diagnosed by the presence of a polypoid mass or a cobblestone appearance on cystoscopy [13]. Being an exophytic inflammatory lesion involving bladder mucosa, polypoid cystitis is histologically characterized by mildly hyperplastic or normal urothelium in the background of a chronically inflamed, congested, and highly edematous stromal tissue. These lesions are often observed on the posterior wall and dome of the bladder, corresponding to localization of the catheter tip [13,15]. In our case, growth arose from the proximal urethra as well as from the bladder neck. One common factor in all cases was a history of long-standing catheterization, which might have predisposed the patient to such a benign lesion but our case was on catheter once for 2 weeks only. In literature, some of the previously reported cases have no relation to the catheter, so the cause in our case can be recurrent asymptomatic cystitis due to inadequate drainage of urine because of obstructive growth. The gross appearance of polypoid cystitis lesions involves multiple small masses, which may be confused with papillary urothelial neoplasm [16], as observed in our case. Moreover, this pathology exhibited signs and symptoms that overlapped with those of urothelial carcinoma. Hence, a definitive diagnosis of polypoid cystitis requires histopathological evaluation [17,18]. In our case, the diagnosis was made following histopathological evaluation.

## 4 Conclusion

Polypoid cystitis is a heterogeneous group of underreported benign urinary bladder conditions that exhibits clinical and radiological features similar to those of urothelial neoplasms. This case is a usual presentation of bladder mass which has an uncommon diagnosis of polypoid cystitis which makes it unusual. Its presentation in the young age group and with urethral involvement is uncommon. It can grow obstructive and can become serious causing retention of urine leading to catheterization or catheterization can itself predispose or not to polypoid cystitis. Though benign but may require aggressive resection and treatment. Adequate histopathological evaluation of these benign lesions is crucial for accurate diagnosis, thus guiding the urologist in selecting an appropriate treatment protocol and follow-up. We should always consider benign causes as differential diagnosis first in young patients without genetic or family history of bladder carcinoma although malignant lesions of bladder are far more common.

## Author contribution

1) Dr. Sajal gupta/SG - concept, writing of manuscript, collection of data.

2) Dr. Prashanth Adiga K/PAK - supervision.

3) Dr. Kishan Raj K/KRK - writing of manuscript, supervision.

4) Dr. Nandakishore B/NKB – supervision.

## Consent

Written informed consent was obtained from the patient for publication and any accompanying images. A copy of the written consent is available for review by the Editor-in-Chief of this journal on request.

## Ethical approval

Yes.

FATHER MULLER TNST|TUT|ONAL ETHTCS COMMTTTEE (FMTEC).

FMIEC/CCMI 801/2024.

## Guarantor

Dr. Sajal Gupta.

## Research registration number

Nil.

## Funding

Nil.

## Conflict of interest statement

Nil.

## References

[1] Jakus D., Jurić I., Šitum M. (2023). Benign urinary bladder masses: rare entities. Afr. J. Urol..

[2] Siraj F., Dulal J., Sharma S., Gupta P., Vasudeva P., Malik A. (2020). Benign mimickers of urinary bladder cancer: a case series. Indian J. Surg. Oncol..

[3] Moch H., Humphery P.A., Ulbright T.M. (2016). WHO Classification of Tumours of the Urinary System and Male Genital Organs.

[4] Dobruch J., Oszczudłowski M. (2021). Bladder cancer: current challenges and future directions. Medicina.

[5] Wentland A.L., Desser T.S., Troxell M.L., Kamaya A. (2019). Bladder cancer and its mimics: a sonographic pictorial review with CT/MR and histologic correlation. Abdom. Radiol..

[6] Kaur S., Gupta A., Gulwani H.V. (2019). A clinicopathological and immunohistochemical study of non-urothelial bladder tumours. Indian J. Cancer.

[7] Hsieh P.F., Chen G.H., Chen S.T., Wu H., Chang C.H., Chou E. (2011). Polypoid cystitis presenting as a protruding urethral mass. Urol. Sci..

[8] Foote F.W., Frazell E.L., Friedman N.B., Ash J.E., Armed Forces Institute of Pathology (1959). Tumors of the Urinary Bladder.

[9] Abu-Yousef M.M., Narayana A.S., Brown R.C. (1984). Catheter-induced cystitis: evaluation by cystosonography. Radiology.

[10] Mostofi F.K. (1954). Potentialities of bladder epithelium. J. Urol..

[11] Lane Z., Epstein J.I. (2008). Polypoid/papillary cystitis: a series of 41 cases misdiagnosed as papillary urothelial neoplasia. Am. J. Surg. Pathol..

[12] Ekelund P., Johansson S. (1979). Polypoid cystitis: a catheter associated lesion of the human bladder. Acta Pathol. Microbiol. Scand. A.

[13] Beeter M.C., Yeh Y.A. Polypoid/papillary cystitis. PathologyOutlines.com website. https://www.pathologyoutlines.com/topic/bladderpolypoidcystitis.html.

[14] Humphrey P.A. (Mar 2013). Polypoid/papillary cystitis. J. Urol..

[15] Voyvoda N., Deveci S., Saglican Y. (2013). A case of polypoid cystitis mimicking bladder tumor in asymptomatic patient. Fırat Tıp Dergisi 2012.

[16] Kim S.H., Yang D.M., Kim N.R. (2004). Polypoid and papillary cystitis mimicking a large transitional carcinoma in a patient without a history of catheterization: computed tomography and magnetic resonance findings. J. Comput. Assist. Tomogr..

[17] Kryvenko O.N., Epstein J.I. (Feb 2019). Mimickers of urothelial neoplasia. Ann. Diagn. Pathol..

[18] Manini C., Angulo J.C., López J.I. (Feb 25 2021). Mimickers of urothelial carcinoma and the approach to differential diagnosis. Clin. Pract..

[19] Sohrabi C., Mathew G., Maria N., Kerwan A., Franchi T., Agha R.A. (2023). The SCARE 2023 guideline: updating consensus Surgical CAse REport (SCARE) guidelines. Int. J. Surg. Lond. Engl..

